# Unsupervised segmentation of noisy electron microscopy images using salient watersheds and region merging

**DOI:** 10.1186/1471-2105-14-294

**Published:** 2013-10-04

**Authors:** Saket Navlakha, Parvez Ahammad, Eugene W Myers

**Affiliations:** 1 School of Computer Science, Machine Learning Department, Carnegie Mellon University, Pittsburgh, PA, USA; 2 Howard Hughes Medical Institute, Janelia Farm Research Campus, Ashburn, VA, USA; 3 Max Planck Institute of Molecular Cell Biology and Genetics, Dresden, Germany

**Keywords:** Image segmentation, Superpixels, Salient watershed, Region merging, Electron microscopy, Unsupervised learning

## Abstract

**Background:**

Segmenting electron microscopy (EM) images of cellular and subcellular processes in the nervous system is a key step in many bioimaging pipelines involving classification and labeling of ultrastructures. However, fully automated techniques to segment images are often susceptible to noise and heterogeneity in EM images (e.g. different histological preparations, different organisms, different brain regions, etc.). Supervised techniques to address this problem are often helpful but require large sets of training data, which are often difficult to obtain in practice, especially across many conditions.

**Results:**

We propose a new, principled unsupervised algorithm to segment EM images using a two-step approach: edge detection via salient watersheds following by robust region merging. We performed experiments to gather EM neuroimages of two organisms (mouse and fruit fly) using different histological preparations and generated manually curated ground-truth segmentations. We compared our algorithm against several state-of-the-art unsupervised segmentation algorithms and found superior performance using two standard measures of under-and over-segmentation error.

**Conclusions:**

Our algorithm is general and may be applicable to other large-scale segmentation problems for bioimages.

## Background

Electron microscopy (EM) images can reveal the physical structure of cellular and subcellular processes in the nervous system at a fine level of resolution. Accurately segmenting such images is a key component of many bioimage related tasks — including labeling, visualization, and classification — in structural biology and neuroscience [1].

However, fully automated methods to segment EM images are computationally challenging to develop due to both natural and synthetic noise in the images and irregularity in cellular structures. Noise can emerge due to variations in histological preparations or in the image acquisition process, or due to natural differences in the brain tissue or organisms of interest. This noise is extremely difficult to overcome experimentally and thus must be accounted for computationally. The physical shape of many structures (e.g. neural membranes) can also vary widely and do not conform to a standard template for detection [2], and intensity and contrast differences may also be equally inconsistent across samples. High-quality EM images can also be very large (millions to tens of millions of pixels), which further constrains the complexity of image processing algorithms. While it may be possible to fine-tune an algorithm to handle nuances within a specific EM preparation, few algorithms have been proposed that can reasonably handle images across a variety of different imaging conditions and preparations. Supervised or semi-supervised techniques are often helpful [3-9], but they require large sets of training data, which are often difficult to obtain in practice, especially across many conditions.

An important initial step of image segmentation is grouping pixels into coherent local regions called *superpixels*. Running algorithms on the decomposed set of superpixels (instead of the original pixels) can aid existing supervised or semi-supervised approaches for EM segmentation as well as other downstream computer vision tasks by simplifying learning and inference. Indeed, in recent years, many unsupervised algorithms have been proposed to generate superpixels and range from graph-based [10-13], to gradient-ascent-basad [14-17], to clustering-based approaches [18] (see Achanta et al. [19] for review). These algorithms have mostly been tailored for processing natural images and are often sensitive to variations in image quality and noise that are inherent to the EM process. These algorithms also employ different constraints and parameters (e.g. different rules to enforce regularity of superpixel size and shape, different measures of superpixel homogeneity, etc.) designed according to their intended application.

**Figure 1:**
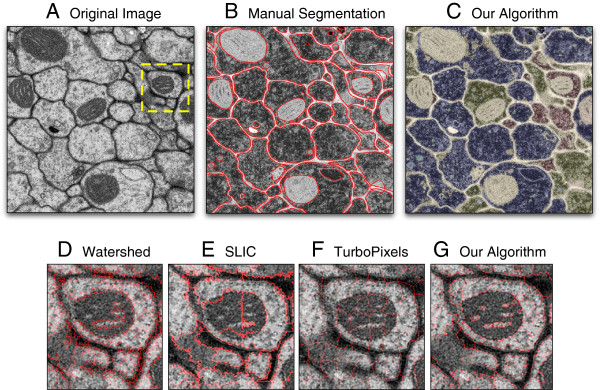
**Overview and example segmentations.** **A)** Original 1000x1000-pixel EM image of the fruit fly ventral nerve cord. **B)** Manual ground-truth segmentation. **C)** The result of our segmentation algorithm after *k*-means clustering. **D–G)** Segmentations of the highlighted region in yellow returned by each algorithm using a total of roughly 1000 superpixels each (boundaries shown in red). Our algorithm better adheres to the true edges compared to Watershed, SLIC, and TurboPixels.

In this paper, we propose a novel, principled unsupervised segmentation algorithm designed specifically to be robust to the types of variation and noise expected in EM images of brain tissue. We propose a two-step approach: First, we develop a novel watershed variant that produces a coarse over-segmentation while strongly preserving edges in the image. This is done by using Canny [20] and probabilistic boundary [21] edges to find high-confidence boundaries, which are then incorporated as constraints into the watershed algorithm. Second, we design a new region merging algorithm to reduce the number of superpixels by merging adjacent regions based on a measure of similarity derived from intensity and texture features. We formalize the merging problem as a graph-theoretic optimization function and use an efficient agglomerative greedy algorithm to find a final partition into the desired number of superpixels. We performed experiments to gather EM images of the fruit fly and mouse nervous systems using two different histological preparations. Using two standard measures of over- and under-segmentation error, we show that our approach offers a significant reduction in the number of superpixels while preserving more true boundaries than existing state-of-the-art algorithms (Figure 1). We also show qualitative results on several additional images. Our results suggest that unsupervised techniques can be used as a general first-pass technique to reduce image complexity without significantly sacrificing accuracy.

## Methods

### The salient watershed algorithm

Given an EM image to segment, the first step is to produce accurate boundary-preserving superpixels. While many algorithms exist for this purpose, the classical watershed algorithm [16] is a natural choice due to its ease of use, efficiency, and scalability. Unfortunately, the standard watershed algorithm suffers from two significant problems: *over-segmentation* and *leakage*. Over-segmentation can usually be corrected with post-processing steps (such as region merging); however, to extract regions from EM images that correspond to precise cellular structures, fixing leakage in the initial segmentation is critical. While dataset-dependent heuristics may help resolve leakage, this does not address the general problem of watershed leakage when segmenting images across different EM preparations and imaging conditions.

**Figure 2:**
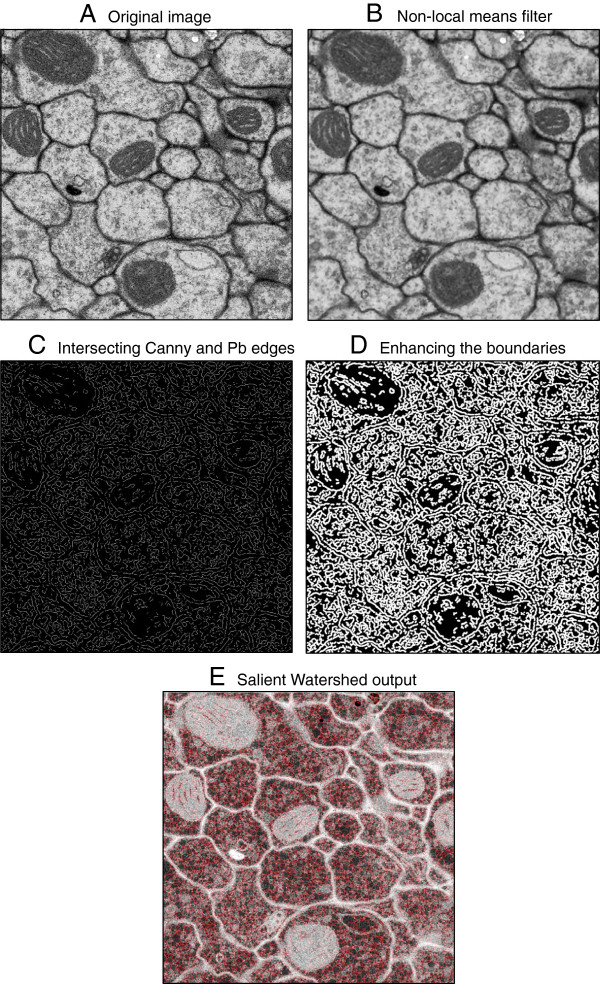
**The Salient Watershed algorithm.** **A)** The original input image. **B)** Non-local means filter applied to de-noise the image. **C)** Detecting high-confidence boundary edges. **D)** Elevating watershed levels where Canny and Pb coincide. **E)** Final watershed output on the enhanced image. See 3 for full description of each step.

To tackle these issues, we propose a novel variant of the watershed algorithm called *Salient Watershed*. The steps of our algorithm are: 

1. **De-noise the image.** We use non-local-means smoothing [22] to both reduce the impact of local noise when detecting boundaries and to reduce unnecessary over-segmentation. In particular, we pre-process the original input image *I* with a 3 × 3 pixel-wide non-local-means filter [22] to obtain *I*_*nl*_ (Figure 2B).

2. **Detect high-confidence boundaries.** First, we apply the Canny edge detector [20] on *I*_*nl*_ to obtain $\nabla I_{\text{nl}}^{\text{canny}}$. Second, we compute the Pb detector [21] on *I*_*nl*_ for a coarse estimate of boundary probabilities $I_{\text{nl}}^{\text{pb}}$, and then we compute an edge map $\nabla I_{\text{nl}}^{\text{pb}}$ by thresholding $I_{\text{nl}}^{\text{pb}}$ at a conservative threshold (1/200). Third, we combine these edges into a hybrid salient edge map via pixel-wise multiplication: $\nabla I_{\text{nl}}^{\text{salient}}=\nabla I_{\text{nl}}^{\text{pb}}.*\nabla I_{\text{nl}}^{\text{canny}}$ (Figure 2C). It has been previously shown that the probabilistic Pb edge detector [21] by itself cannot adequately segment EM images without re-training on specific type of images [5]. Combining the Canny and Pb boundary detectors gives us the ability to find high-likelihood salient boundaries that retain precise edge localization without resorting to parameter tuning or re-training for different kinds of tissue samples.

3. **Elevate watershed levels where Canny and Pb coincide.** Next, we compute the Euclidean distance transform on $\nabla I_{\text{nl}}^{\text{salient}}$ to obtain $I_{\text{dist}}^{\text{salient}}$ and then compute an enhanced edge map $I^{\text{enhance}}=e^{-2*I_{\text{dist}}^{\text{salient}}}$ (Figure 2D). This step elevates the watershed along the intersection of Canny and Pb lines and provides an exponential fall-off as the distance to these lines increases. It also helps bridge small gaps that may exist in the boundaries.

4. **Run watershed on the enhanced image.** Finally, we apply the classical watershed algorithm on *I*^*enhance*^ to obtain the final over-segmented image $I_{\text{Ws}}^{\text{enhance}}$ (Figure 2E).

By incorporating the notion of edge saliency into the watershed computation, we ensure that salient boundaries are preserved. This addresses the leakage problem consistently. While this procedure adds additional computational complexity to the original watershed procedure, *Salient Watershed* is a more robust algorithm that can be applied to many EM datasets to produce a first-pass segmentation without tuning parameters.

This algorithm produces an initial (over-segmented) set of superpixels (regions), which are then further collapsed using an agglomerative merging algorithm, as described below.

### The region merging algorithm

Region merging is often performed after superpixels are generated to collapse neighboring regions. There are three aspects to region merging: the features used to represent each region, a measure of similarity between regions in feature-space, and an objective function for merging regions. We describe each of these aspects below.

Each region is defined by a normalized intensity histogram and a set of normalized texture histograms computed using pixel values in the region. Texture is an important cue used by humans when manually segmenting and annotating EM images [23], and its use has become popular in many computer vision tasks today [24]. Varma and Zisserman [25] proposed an effective set of 38 filters (6 orientations × 3 scales × 2 oriented filters + 2 isotropic filters), but only recorded the maximum filter response across each orientation, leading to 8 total filter responses at each pixel. Each region is thus represented by a *b* × 9 feature matrix, where *b* = 32 is the number of bins in each histogram.

Most previous approaches compute the similarity between two regions in feature-space based on the Euclidean or Manhattan distances [26], by comparing means and standard deviations of feature vectors [27,28], or using information-theoretic measures [29]. The downside of these measures is that they treat each histogram bin independently and, as a result, two histograms that differ slightly in adjacent bins are treated as equally distant as two histograms that differ equally in far-apart bins. To avoid this problem, we use the Earth Mover’s Distance (EMD) [30], which computes the minimum cost to transform one histogram to exactly match the other using transformation costs that depend on the linear distance between bins. EMD can be solved quickly using a constrained bipartite network flow routine [31]. Overall, the similarity between two adjacent regions *r* and *r*^′^ is defined as:

(1) $$\begin{array}{lll} \;s(r,r^{\prime }) & =\exp(-\min(r_{\text{size}},r_{\text{size}}^{\prime }))+\exp(-\text{EMD}({\text{Int}}_{r},{\text{Int}}_{r^{\prime }})\; & \;\\ & \;-\alpha \overset{8}{\underset{i=1}{\sum }}\text{EMD}({\text{Text}}_{r,i},{\text{Text}}_{r^{\prime },i})),\; \end{array}$$

where the first term biases towards collapsing smaller regions; Int_*r*_ is the normalized intensity histogram of region *r*; Text_*r*,*i*_ is the *i*^*th*^ normalized texture histogram of region *r*; and *α* is a parameter to weigh the contribution of the texture component (we set *α* = 1/8). We use EMD to compute the similarity between both normalized features (intensity and texture), and thus born terms lie on roughly the same scale.

The final aspect of the algorithm is the merging optimization function [26]. We define a predicate  that states that every region *r* should be "sufficiently" different compared to each of its neighbors. Formally:

(2) $$\begin{array}{l} P(r)=\left\{\begin{array}{ll} \text{true} & \;\text{if}\;s(r,r^{\prime })\leq \tau ,\forall r^{\prime }\in N(r)\\ \text{false} & \;\text{otherwise}, \end{array}\right) \end{array}$$

**Algorithm 1** Region-Merging(I,L,NumSPs)

where *N*(*r*) are the regions adjacent to *r*. If this statement is true for region *r*, we call *r* an "island". We seek to find a segmentation such that  holds for every region. In graph-theoretic terms, we start with the region adjacency graph *G* = (*V*,*E*), defined by nodes *V* (regions) and with edges *E* connecting adjacent regions. To merge two regions means to contract the edge between them; our goal is thus to find a set of edges whose contraction results in a graph satisfying  for every region. We find such a set using a greedy agglomerative algorithm: we start with the regions produced by the *Salient Watershed* algorithm, and iteratively merge the pair of neighboring regions that are most similar. This process can stop either when the similarity between any two adjacent regions is < *τ* (at which point every region is guaranteed to be an island according to *τ*) or when the desired number of superpixels is met (as we do here). Pseudocode of the region merging algorithm is shown in Algorithm 1.

### Comparing segmentations versus ground-truth

To evaluate performance, we performed experiments and collected three 1000  × 1000-pixel EM images of the nervous system: 2 images were from the fruit fly ventral nerve cord fixed using a high pressure freezing (HPF) protocol, and 1 image was from the mouse cortex using a perfusion DAB-based protocol (e.g. [32]). We manually segmented membranes, mitochondria, and other neuronal structures in these images (Figure 1A and 1B) and extracted ground-truth boundary matrices for each. We also collected two additional images of the mouse cortex using HPF, which we analyzed qualitatively.

To compare an algorithm’s segmentation *P* with the ground-truth *Q*, we use two standard metrics: the asymmetric partition distance (APD) and the symmetric partition distance (SPD) [33]. APD(*P*,*Q*) computes, over all regions *r* ∈ *P*, the maximum percentage of pixels in *r* that map onto a single ground-truth segment. SPD(*P*,*Q*) finds the maximal matching between regions in *P* and *Q* and computes the overall percentage of pixels that must be deleted from both images in order to make each pair of matched regions equivalent. APD penalizes "spill-over" of segments across ground-truth boundaries, but does not penalize over-segmentation. On the other hand, SPD measures exact 1-1 correspondence between segmentations and does penalize over-segmentation. We report 1- SPD(*P*,*Q*) as a percentage, so in both measures higher percentages are better.

## Results and discussion

We compared our algorithm against TurboPixels [17] and a MATLAB implementation of SLIC [19,34]. TurboPixels uses geometric flows to find regions that are approximately uniform in size and shape while also preserving smooth boundary edges, and it is specifically designed to produce high-quality over-segmentations. SLIC is a clustering method based on *k*-means that was shown to be superior to several graph-based and gradient-ascent-based algorithms on segmenting mitochondria in EM images [18]. It was also recently shown in a large-scale comparison to be amongst the best performing algorithms on the Berkeley segmentation dataset [19], and thus represents the current state-of-the-art. We ran each algorithm on our EM images and varied the number of superpixels returned by adjusting parameters in the algorithm. For each segmentation, we computed the over- and under-segmentation error (SPD and APD, respectively).

**Figure 3:**
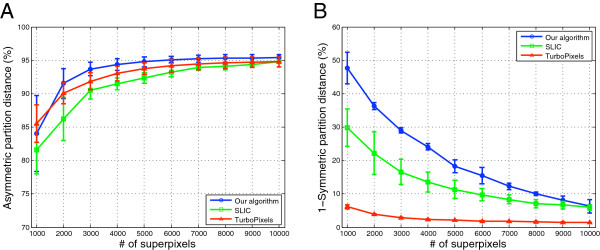
**Under- and over-segmentation error of each algorithm with respect to ground-truth.** The average and standard deviation of **A)** APD and **B)** SPD for each algorithm on our EM benchmark dataset. Overall, our algorithm preserves more true ground-truth boundaries (APD) and better captures true ground-truth segments within a single region (SPD) compared to TurboPixels and SLIC.

Our algorithm more strictly adheres to true boundaries compared to the other algorithms across nearly the entire range of superpixels (Figure 1D–G and Figure 3A). For example, at roughly 2000 superpixels on the first fruit fly image, our algorithm has an APD of 93.72% compared to 88.98% for TurboPixels and 86.89% for SLIC. Thus, we can achieve over three orders of magnitude reduction in the number of superpixels (compared to the original image) while still preserving over 90% of the true boundaries. Some predicted boundary contours may indeed be correct but do not align exactly with the ground-truth boundaries; thus, this value actually represents a lower-bound on performance. In practice, over-segmentation is often more permissive than under-segmentation because it is relatively easy for downstream analyses to specify additional merges (e.g. via classification) but more difficult and labor-intensive to reconstruct a lost boundary.

Our algorithm also outperforms the other methods in extracting true regions in their entirety (Figure 3B). The SPD penalizes over-segmentation and measures exact concordance between the ground-truth and algorithm partitions. At 1000 superpixels, our algorithm has an average SPD of almost 50% compared to 7% (TurboPixels) and 30% (SLIC). This means that half the pixels in our partition are *exactly* matched to ground-truth regions. Our ground-truth was constructed to consider entire membranes as single regions (as a biologist might), but there may be small substructures within membranes that persist due their markedly different features. These regions will naturally be left unmerged by each algorithm; a more fine-grained ground-truth segmentation would thus increase these percentages further. TurboPixels especially suffers on the SPD measure because it generates regular- and grid-like superpixels (Figure 1F); EM images, however, contain many irregularly-shaped structures that do not fit this mold.

Our algorithm and SLIC perform similarly under both metrics when the number of desired superpixels is large (Figure 3 at 10,000 superpixels), but diverge as fewer superpixels are requested. This suggests that both methods may be comparable at high numbers of superpixels, but that our region merging algorithm is more robust at preserving boundaries than the clustering-based approach used by SLIC.

We also compared our *Salient Watershed* algorithm to the classical watershed algorithm [16]. On the first image, for example, the latter produced a segmentation with 43,252 regions and an APD of 94.17%. *Salient Watershed* produced a segmentation with 13,252 regions and an APD of 95.25%. APD can not increase with subsequent merges; the fact that our segmentation produces a *higher* APD with more than 3x *fewer* regions testifies to the strong edge-preserving property of our salient watersheds.

Next, to determine whether our superpixels may be used for classification, we took the 1000 superpixels generated by our algorithm and clustered them in feature-space using *k*-means (Figure 1C). Co-clustered regions were assigned the same color (we used *k* = 13 but found similar results for many *k*). Visual inspection shows that indeed many similar structures — in particular mitochondria (light green) and membranes (purple) — are similarly colored. This implies that the superpixels that comprise these regions represent homogeneous biologically structures and that they are well-separated by intervening boundaries in feature space. This clustering represent a first-pass unsupervised labeling of EM images that can be further improved via supervised techniques [3,5].

**Figure 4:**
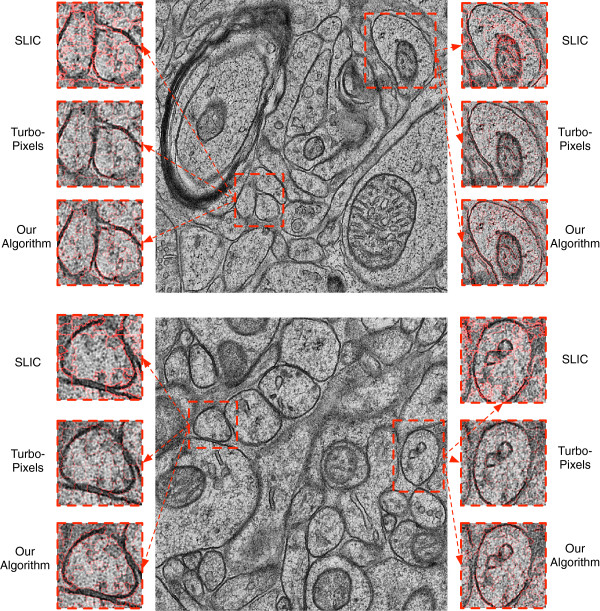
**Qualitative results on two additional images.** We ran SLIC, TurboPixels, and our algorithm on two additional images of the mouse cortex prepared using a high-pressure freezing EM protocol. Our approach again preserves boundaries and edges with more fidelity than the other methods, despite no adjustment of parameters.

Finally, we demonstrate the performance of our algorithm versus SLIC and TurboPixels qualitatively on two additional images of the mouse cortex prepared using high pressure freezing (Figure 4). The previous images of the mouse cortex were obtained using DAB. Without altering *any* parameters, we ran each algorithm using 2,000 superpixels and visually compared the predicted boundaries. As with the previous images, our method preserves intricate membrane boundaries much better than the other techniques and produces more homogeneous regions. We also find superior performance when capturing irregularly-shaped regions, and we are better able to separate regions that are separated by a thin boundary (e.g. two membrane boundaries that lie adjacent to one another; Figure 4). Both of these types of heterogeneity are widespread in EM images and not easily captured by methods that make assumptions about edge properties or the distribution of noise in EM images [28]. This further suggests that our unsupervised approach is robust to some natural variations caused by different histological preparations in EM neuroimages.

## Conclusions

Accurately segmenting electron microscopy images is an important problem for many neuroimage related tasks, but it also presents several computational challenges due to the noise and variation inherent in tissue samples and in the EM chemistry and image acquisition processes. We presented an unsupervised algorithm to generate boundary-preserving superpixels by combining a salient watershed algorithm with robust region merging. On a benchmark dataset of noisy EM images, our algorithm outperformed two state-of-the-art methods using two standard measures of over- and under-segmentation error. While our method has additional computational complexity, we place emphasis on accuracy and contend that downstream time spent in EM image analysis will be reduced through more accurate segmentations.

While aspects of this general pipeline for segmentation (edge detection, watershed, region merging) have been used in previous works [8,9,28], the specific sequence of steps as outlined in this paper is novel. This combination of components offers our unsupervised approach a level of generality and robustness that can handle many types of noise present in heterogeneous EM data. Our approach also uses few parameters and may be usable across different EM histological preparations and for other large-scale bioimage segmentation problems (e.g. segmentation of cells, nuclei, or proteins within fluorescence microscopy images).

## Competing interests

The authors declare that they have no competing interests.

## Authors’ contributions

The project was conceived by PA and EWM. PA and SN designed the algorithms and wrote the software. SN generated the ground-truth data and performed the quantitative evaluations. SN, PA and EWM wrote the paper. All authors read and approved the final manuscript.
